# First-Year Real-Life Experience with Intravitreal Faricimab for Refractory Neovascular Age-Related Macular Degeneration

**DOI:** 10.3390/pharmaceutics16040470

**Published:** 2024-03-27

**Authors:** Wissam Aljundi, Loay Daas, Shady Suffo, Berthold Seitz, Alaa Din Abdin

**Affiliations:** Department of Ophthalmology, Saarland University Medical Center UKS, 66421 Homburg/Saar, Germany; loay.daas@uks.eu (L.D.); shady.suffo@uks.eu (S.S.); berthold.seitz@uks.eu (B.S.); alaadin.abdin@uks.eu (A.D.A.)

**Keywords:** faricimab, anti-VEGF, Ang-2, age-related macular degeneration, subretinal fluid

## Abstract

**Background**: To evaluate the outcomes of intravitreal faricimab (IVF) for refractory neovascular age-related macular degeneration (nAMD) and investigate the impact of baseline optical coherence tomography, biomarkers for total IVF injections are needed. **Methods**: A retrospective analysis of 33 eyes of patients who completed one year (52 W) of treatment with IVF. The eyes received four IVF injections (6 mg/0.05 mL) as the upload phase. Thereafter, the treatment interval was extended to 8 or 12 weeks if disease activity was not recorded. The outcome measures included best-corrected visual acuity (BCVA), central macular thickness (CMT), subfoveal choroidal thickness (SFCT), and retinal fluid distribution. **Results**: A total of 33 eyes were included. CMT decreased significantly at 52 W (*p* < 0.01). BCVA and SFCT did not change significantly at 52 W (*p* > 0.05). The number of eyes with subretinal fluid decreased significantly at 52 W (*p* < 0.01). Complete fluid resolution was achieved in 20 eyes (60%). The total number of injections was significantly negatively correlated with the presence of hyperreflective dots at baseline (HRDs, *p* < 0.01) and SFCT at baseline (*p* < 0.01). **Conclusions**: IVF led to a significant reduction in CMT with stabilization of BCVA. The total number of injections was lower in eyes with HRDs and increased SFCT at baseline. This might provide clues regarding response to IVF for future studies.

## 1. Introduction

Age-related macular degeneration (AMD) represents the most common cause of severe vision loss and blindness in Europe. According to recent estimates, more than 200 million people worldwide are affected by AMD. This number is expected to rise to 288 million by 2040 [1]. AMD is classified based on clinical findings as early, intermediate, and advanced, which has two forms: the slowly progressive dry form and the rapidly progressive exudative form. Vision-related quality of life is severely impaired in all types of AMD. Moreover, this effect was even more severe in patients with exudative AMD in at least one eye [2,3]. Macular neovascularization (MNV) is the hallmark of exudative “neovascular” AMD (nAMD) and is responsible for approximately 90% of severe vision loss in patients with AMD. Vascular endothelial growth factor (VEGF) is known to play an important role in the regulation of neovascularization (NV) and vascular permeability [4]. The introduction of anti-VEGF drugs has significantly reduced the prevalence and severity of vision loss in patients with nAMD [5]. However, these drugs cause several dysfunction phenomena such as non-response, recurrence, and resistance [6,7]. This has led to an urgent demand to develop new therapeutic targets and strategies to avoid these phenomena and prompted the consideration that factors other than VEGF are also involved in the pathogenesis of nAMD.

The novel humanized bispecific monoclonal immunoglobulin G1 antibody “Faricimab” was approved for the treatment of nAMD in Europe and the USA in 2022 [8]. Faricimab derives its significance from its mechanism of action, as it binds to and inhibits both VEGF-A and angiopoietin-2 (Ang-2) [9]. Ang-2 is thought to contribute significantly to the development of MNV, inflammation, and vascular destabilization through tyrosine kinase with immunoglobulin and the epidermal growth factor homology domain 2 (Tie-2) pathway. Angiopoietins bind to Tie-2 endothelial receptors and thereby contribute to the regulation of vasculogenesis [10]. Ang-1 and Ang-2 bind competitively to Tie-2. The binding of Ang-1 to Tie-2 activates downstream signaling pathways that ultimately maintain vascular stability and inhibit vascular permeability. The binding of Ang-2 to Tie-2 has the opposite effect: it deactivates the signaling pathway and decreases vascular stability. Ang-2 is known to be increased by ischemia or oxidative stress (as in AMD).

Moreover, Ang-2 has been shown to increase vascular sensitization to VEGF. Therefore, inhibition of this pathway would potentially have synergistic effects with anti-VEGF agents by further reducing vascular permeability and inflammation compared to anti-VEGF alone, and thus have a stronger therapeutic effect [11,12,13].

It is well known that optical coherence tomography (OCT) represents an essential tool for the diagnosis and follow-up of patients with AMD nowadays. Not only is it a widely available, user-friendly, fast and non-invasive device, but it provides high-resolution in vivo imaging of the chorioretinal anatomy and vasculature. In addition, OCT offers a variety of biomarkers that could provide insights into treatment outcome and prognosis in patients with different macular diseases, such as AMD [14]. For example, Kim et al. found that the presence of prechoroidal clefts (PCCs) was associated with worse visual outcome in patients with nAMD [15]. Similarly, Pokroy et al. reported that the presence of subretinal hyperreflective material (SHRM) also served as a negative prognostic factor of visual outcome under treatment with bevacizumab in eyes with nAMD [16].

In this study, our main aim was to evaluate the first-year outcome of intravitreal faricimab (IVF) in patients with refractory AMD in a real-life setting. In addition, we investigated the impact of a variety of baseline biomarkers on the total number of injections at the end of the first year.

## 2. Methods

### 2.1. Study Design

In this retrospective monocenter study, 33 eyes of 33 patients with refractory nAMD were included and followed for 12 months after first IVF injection (52 W).

We defined a refractory nAMD as persistent intraretinal fluid (IRF) and/or subretinal fluid (SRF) and/or retinal pigment epithelial detachment (PED) despite treatment with at least two anti-VEGFs as assessed using spectral-domain optical coherence tomography (SD-OCT).

We considered the day of the first IVF as the baseline for the follow-up. All outcomes were evaluated at baseline, and then every 4 weeks until week 52.

SD-OCT examinations of all patients at each follow-up were reviewed by 2 retinal specialists.

We performed 4× IVF 6 mg/0.05 mL every 4 weeks as an upload dose. Thereafter, the treatment interval was extended to 8 or 12 weeks if disease activity was not recorded. The criteria for re-injection after the upload phase were increase in and/or persistence of fluids, increase in CMT by at least 100 µm, and new MNV or new macular hemorrhages [17]. All eyes were switched to IVF after treatment with at least two other anti-VEGF agents, including bevacizumab, ranibizumab, and aflibercept. Eyes treated with intravitreal brolucizumab were not included in this study.

### 2.2. Performing the Intravitreal Injections

All IVIs were performed in a designated center for intravitreal injections in our Department of Ophthalmology at Saarland University Medical Center and in accordance with the local guidelines in Germany [18,19]. The intravitreal injections were performed as an outpatient procedure under sterile conditions in an operating room suitable for intraocular surgery in order to reduce the risk of endophthalmitis, which represents the most serious complication [20]. The injections were performed 3.5–4.0 mm posterior to the limbus using an injection cannula of 30-gauge diameter and 10 mm length between the horizontal and vertical rectus muscles via pars plana. Quadrant selection was decided by the microsurgeon. The injection was performed after displacement of the conjunctiva and gradual injection perpendicular to the sclera in order to minimize the risk of endophthalmitis and drug reflux as well as to avoid an injury to the lens or retina [21,22].

### 2.3. Inclusion and Exclusion Criteria

The inclusion criteria:

- Eyes with refractory nAMD (as defined above).
- A minimum follow-up of 52 weeks.

The exclusion criteria:

- History of treatments with photodynamic therapy (PDT).
- Coexisting vitreoretinal pathology.
- Naïve patients.

### 2.4. Outcome Measures

- Best-corrected visual acuity (BCVA) assessed in decimals and converted to logMAR.
- Central macular thickness (CMT) as assessed with the Spectralis SD-OCT device (Heidelberg Engineering, Heidelberg, Germany) and defined as mean retinal thickness (µm) between internal limiting membrane (ILM) and Bruch’s membrane (BM) in the central 1 mm of the fovea.
- Subfoveal choroidal thickness (SFCT) measured by enhanced-depth imaging OCT (EDI-OCT) and defined as the vertical distance between the hyperreflective line of BM and the hyperreflective line of the inner surface of the sclera [23]. EDI-OCT images were taken by different technicians and analyzed in a masked manner.
- Changes in retinal fluid distribution.
- Total number of IVF injections performed at 52 W.
- Impact of various baseline SD-OCT biomarkers on total number of IVF injections.

### 2.5. Analysis of Baseline SD-OCT Biomarkers

**Figure 1:**
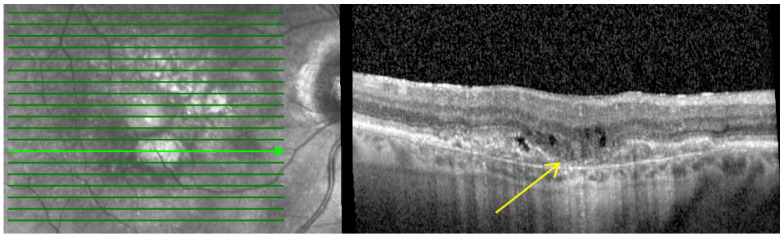
Baseline spectral-domain optical coherence tomography image representing macular atrophy (arrow).

**Figure 2:**
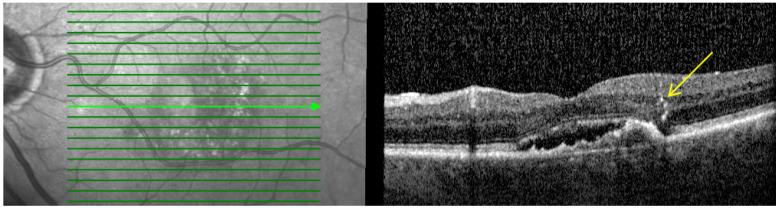
Baseline spectral-domain optical coherence tomography image representing hyperreflective dots (arrow).

**Figure 3:**
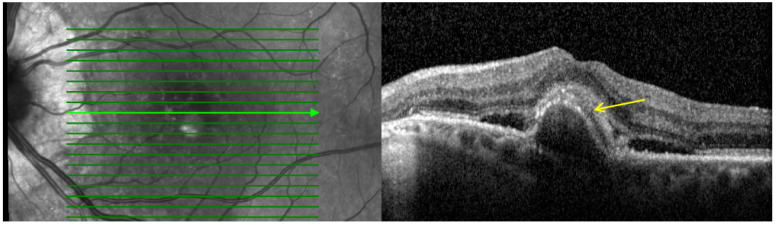
Baseline spectral-domain optical coherence tomography image representing subretinal hyperreflective material (arrow).

**Figure 4:**
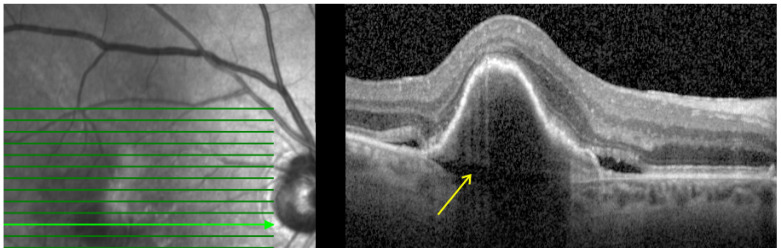
Baseline spectral-domain optical coherence tomography image representing prechoroidal cleft (arrow).

The following biomarkers were recorded and compared between baseline and last follow-up (52 W) in order to investigate the impact on the final total number of IVF injections performed and thus potentially on the treatment response:

1. **Macular atrophy (MA):** Characterized by in-lesion photoreceptor death and visual impairment and typically follows progressive atrophy and thinning of the retinal pigment epithelium (RPE) and choriocapillaris. The loss of photoreceptors can be detected by the thinning of the Henle fiber layer. This could be detected by OCT by features such as loss of the ellipsoid layer and outer limiting membrane as well as thinning of the outer nuclear layer, which together with the Henle fiber layer and photoreceptors appear as a single hyporeflective band on OCT images as shown in Figure 1 [24,25,26].

2. **Hyperreflective dots (HRDs):** Defined as small, well-circumscribed lesions with a reflectivity equal to or greater than the RPE as shown in Figure 2. These lesions often occur over drusen and are associated with delays in visual acuity. It is hypothesized that reduced oxygen supply promotes anterior migration of RPE cells, which manifests as HRDs. On the other hand, HRDs in nAMD are also thought to be microglia, common immune cells in the inner retina that migrate from the inner retina to the outer retina when activated in an environment associated with degeneration [27,28].

3. **Subretinal hyperreflective material (SHRM):** Defined as a hyperreflective material located between the neurosensory retina and RPE as shown in Figure 3. In eyes with nAMD, SHRM is common and often persists after anti-VEGF treatment. SHRM is thought to have a negative impact on visual acuity and is likely to be composed of fluid, fibrin, blood, scar tissue, and MNV [29,30,31].

4. **Prechoroidal cleft (PCC):** Defined as hyporeflective space between the RPE fibrovascular tissue and Bruch membrane as shown in Figure 4. PCCs are closely associated with a poor visual prognosis and increased risk of submacular hemorrhage or RPE tear [15]. The origin of the cleft has been attributed to a possible accumulation of the fluid generated by the fibrovascular tissue. However, its correlation with lesion activity and treatment remains unknown [32].

### 2.6. Statistical Analysis

Data were collected using Microsoft Excel 2010 (Microsoft Corporation, Redmond, WA, USA) and analyzed using SPSS version 27 (SPSS Inc., Chicago, IL, USA). To compare means, we used the paired samples test after performing Bonferroni correction. We used the chi-square (χ^2^) test to compare categorical variables. Correlations were tested with Pearson’s correlation coefficient (r). The level of statistical significance was set at *p* < 0.05 with a 95% confidence interval.

## 3. Results

The patients’ baseline characteristics are listed in Table 1.

No intraocular inflammatory events were recorded during follow-up.

Changes in best-corrected visual acuity (BCVA, logMAR) during follow-up are listed in Table 2.

Changes in central macular thickness (CMT, µm) during follow-up are listed in Table 3.

Changes in subfoveal choroidal thickness (SFCT, µm) during follow-up are listed in Table 4.

The changes in retinal fluid distribution and SD-OCT biomarkers during follow-up are listed in Table 5.

## 4. Discussion

Faricimab is a novel, 150 kDa size, bispecific antibody, which binds to and blocks both VEGF-A and Ang-2 simultaneously. The role of the Tie-2 pathway in the development of MNV has recently been intensively investigated [11,12]. To the best of our knowledge, this paper reports one-year real-life experience with IVF for refractory nAMD for the first time.

Despite the fact that AMD is not considered a classical inflammatory disease, it is known that inflammatory cells are involved in both the development and progression of nAMD. Both macrophages and giant cells were identified around drusen, at the breakdown of Bruch’s membrane as well as in MNV membranes [33]. An additional inflammatory cellular involvement has been identified with macrophage-derived cytokines such as tumor necrosis factor-alpha (TNF-a) and IL-1, which promote the expression of intercellular adhesion molecule-1 (ICAM-1) in the RPE and vascular endothelial cells. In addition, MNV could be enhanced through the action of macrophage-derived cytokines that promote the proliferation and migration of vascular endothelial cells [33,34,35]. Furthermore, microglial cells are known to play a role in the pathogenesis of nAMD, as activated microglia are found in the outer retina and subretinal regions of eyes with nAMD [36]. After being activated by retinal injury and degeneration, microglial cells migrate to the injured outer retina, facilitating phagocytosis of debris and producing various proinflammatory cytokines and chemokines. This results in a neurotoxic milieu leading to disease progression [33,36]. In patients with refractory nAMD, chronic inflammation may induce permanent structural damage to the vascular walls of the MNV complex, potentially causing permanent pathological vascular permeability and persistent exudation that is not responsive to classical anti-VEGF therapy [37]. In addition, inflammatory stimulation could enhance fibrosis of the MNV, which acts as a resorption barrier reducing the sensitivity to anti-VEGF drugs [38]. These aforementioned considerations justify the efforts to target the inflammatory process in nAMD, especially to avoid severe vision loss due to late disease manifestations such as scarring and fibrosis [39,40].

In nAMD, chronic inflammation often results in the release of stromal and immune cells, which initiate the transition of the neovascular endothelial bundle to a fibrovascular membrane [39]. Excessive fibrosis leads to subretinal scarring with irreversible loss of photoreceptors, RPE cells, and choroidal blood vessels, causing the majority of severe vision loss in patients with nAMD. Subretinal fibrosis appears in half of patients with nAMD receiving ongoing anti-VEGF therapy, and its pathogenesis is poorly understood [41]. As mentioned above, activated microglia contribute to the inflammatory process by expressing cytokines such as Interleukin-6 (IL-6) and TNF-a, which can exacerbate fibrosis. These cytokines, especially IL-6, represent a key mediator promoting subretinal fibrosis. Therefore, targeting inflammation by blocking Ang-2 and the corresponding pathway would be a potential therapeutic approach not only for macular neovascularization, but also for preventing subretinal fibrosis [40,42,43]. Furthermore, in a recent study, Linder et al. found that simultaneous Ang-2/VEGF inhibition prevented the progression of subretinal fibrosis in preclinical mouse models of MNV [44]. These facts provide positive evidence for the possible role of dual Ang-2/VEGF inhibition in preventing subretinal fibrosis. However, larger studies with more patients and longer follow-up are needed to confirm this consideration.

We found that treatment with IVF in eyes with refractory nAMD resulted in significant anatomical improvement with sustained visual acuity. This corresponds to the results of previously published short-term papers listed in Table 6 [45,46,47,48]. The reason for the statistically non-significant improvement in BCVA in both our current study and the papers listed in Table 6 could be attributed to the fact that the included eyes were pretreated with various anti-VEGF agents, which might suggest the presence of both RPE and photoreceptor atrophy, preventing a major and rapid improvement of BCVA [26].

The development of MA in nAMD and under anti-VEGF treatment is described in the literature [26,49,50]. Two main factors contribute to MA. First, the natural history of nAMD and the apoptosis of RPE cells. Second, treatment with anti-VEGF leads to a reduced choroidal and retinal vascular perfusion. This increases retinal ischemia and leads to MA [49,50].

Our recent study found that patients with MA had shown significantly lower improvement in BCVA. The generally worse outcome of anti-VEGF therapy in eyes with pre-existing MA is supported by previously published papers [51,52].

Moreover, we found that SFCT correlated negatively with the total number of IVF injections. The impact of choroidal thickness on the treatment outcome of nAMD has only been limitedly reported in the literature. Kang et al. found that SFCT was a predictor of favorable response and BCVA improvement during treatment with ranibizumab in 40 eyes with nAMD [53]. In addition, Beraldo et al. found that choroidal thickness had a positive impact on response to aflibercept in 15 naïve patients with nAMD. Patients with increased choroidal thickness at baseline had experienced the highest improvement in BCVA and greater reduction in CMT. One of the possible explanations of this positive effect could be due to retained choriocapillaris [54]. Our research group previously found that a thicker choroid at baseline seems to have a positive impact on long-term outcomes of treatment with anti-VEGF agents, especially in eyes with MNV-1 [55].

Recently, Kataoka et al. analyzed the 6-month results of switching from aflibercept to faricimab in 130 eyes with refractory nAMD. Of these eyes, only 53 (40%) achieved a follow-up time of 6 months. Similar to our study, no significant differences in SFCT were observed in these eyes after switching to IVF during follow-up [48].

Furthermore, treatment intervals for IVF could be extended to 16 weeks, which consequently would mean a lower number of injections for each patient per year [9]. This could be of great importance for both patients and healthcare systems as it results in lower therapeutic costs and complication rates. Moreover, a large metanalysis estimated the rate of reported non-adherence in nAMD at about 60% at 24 months of follow-up [56]. In our study, IVF led to a significant reduction in total intravitreal injections needed per year. This might be crucial for patients with refractory nAMD to reduce the possibility of non-adherence due to the long-term burden of this chronic disease. Therefore, the significant reduction in the number of injections used at the end of the first year, from 8.6 ± 1.2 to 7.1 ± 2.4 IVIs/eye, represents a major outcome, despite the fact that no significant increase in BCVA was achieved among our refractory cases under treatment with IVF.

HRDs have been recognized as structural biomarkers for disease progression, prognosis, and response to treatment in a variety of retinal diseases such as AMD [57], diabetic macular edema [58], and central serous chorioretinopathy [59]. The origin of HRDs is not yet fully understood; however, several studies have suggested that HRDs occur as accumulations of activated microglial cells in the retina and may represent an in vivo biomarker of local inflammation. Mao et al. found that the concentrations of VEGF and IL-10 in the aqueous humor were significantly increased in nAMD patients with HRDs compared to patients without HRDs. Furthermore, the VEGF concentration in the aqueous humor correlated positively with the number of HRDs. In addition, the levels of VEGF in the aqueous humor were positively correlated with the number of HRDs.

After anti-VEGF treatment, patients with HRDs in nAMD had a worse visual gain but a significant decrease in CMT [60].

We found that eyes with HRDs at baseline required a significantly lower total number of IVF injections, which may reflect a favorable response to IVF. This efficacy of faricimab in eyes with HRDs at baseline could be explained by its mechanism of action, as faricimab actively inhibits inflammation by inhibiting Ang-2. This may present IVF as a promising therapeutic option in patients with refractory nAMD and HRDs.

## 5. Conclusions

IVF led to a significant reduction in CMT with stabilization of BCVA at the end of the first year of treatment. The total number of injections performed was lower in eyes with HRDs and increased SFCT at baseline. This might provide clues regarding response to IVF. However, multicentric prospective studies with larger numbers of patients are still needed.

## Figures and Tables

**Table 1:** Baseline characteristics.

Age in years	82 ± 5
Gender (Male:Female)	55%:45%
Previous total intravitreal injections	44 ± 21
Previous intravitreal bevacizumab	12 ± 10
Previous intravitreal ranibizumab	11 ± 9
Previous intravitreal aflibercept	21 ± 16
Previous injections in previous year before switching to IVF	8.6 ± 1.2
BCVA (logMAR)	0.73 ± 0.18
CMT (µm)	368 ± 34
SFCT (µm)	171 ± 64
Type of MNV (1:2:3)	72%:22%:6%

BCVA: best-corrected visual acuity (logMAR). CMT: central macular thickness (µm). SFCT: subfoveal choroidal thickness (µm). MNV: macular neovascularization. The total number of performed IVIs per eye decreased significantly from 8.6 ± 1.2 IVIs in the previous year before switching to IVF to 7.1 ± 2.4 IVIs at 52 W (*p* < 0.01).

**Table 2:** Changes in best-corrected visual acuity (BCVA, logMAR) *.

0 W	4 W	8 W	12 W	16 W
0.73 ± 0.18	0.74 ± 0.16 (*p* = 0.30)	0.73 ± 0.16 (*p* = 0.89)	0.74 ± 0.18 (*p* = 0.36)	0.74 ± 0.21 (*p* = 0.57)
20 W	24 W	28 W	32 W	36 W
0.72 ± 0.21 (*p* = 0.77)	0.69 ± 0.21 (*p* = 0.14)	0.68 ± 0.20 (*p* = 0.14)	0.66 ± 0.20 (*p* = 0.07)	0.65 ± 0.22 (*p* = 0.07)
40 W	44 W	48 W	52 W	∆BCVA
0.67 ± 0.23 (*p* = 0.24)	0.66 ± 0.24 (*p* = 0.11)	0.66 ± 0.26 (*p* = 0.15)	0.68 ± 0.20 (*p* = 0.06)	−0.04 ± 0.14

* Results reported as mean ± SD. The *p*-values shown refer to the comparison with baseline using paired samples test after performing Bonferroni correction.

**Table 3:** Changes in Central Macular Thickness (CMT, µm) *.

0 W	4 W	8 W	12 W	16 W
368 ± 34	371 ± 69 (*p* = 0.86)	357 ± 76 (*p* = 0.17)	333 ± 61 (*p* = 0.03)	327 ± 66 (*p* = 0.01)
20 W	24 W	28 W	32 W	36 W
314 ± 69 (*p* < 0.01)	333 ± 76 (*p* = 0.01)	318 ± 68 (*p* < 0.01)	325 ± 62 (*p* = 0.02)	325 ± 57 (*p* < 0.01)
40 W	44 W	48 W	52 W	∆CMT
321 ± 61 (*p* = 0.02)	327 ± 59 (*p* < 0.01)	305 ± 53 (*p* < 0.01)	280 ± 67 (*p* < 0.01)	−87 ± 95

* Results reported as mean ± SD. *p*-values shown refer to the comparison with baseline using paired samples test after performing Bonferroni correction.

**Table 4:** Changes in Subfoveal Choroidal Thickness (SFCT, µm) *.

0 W	4 W	8 W	12 W	16 W
171 ± 64	171 ± 63 (*p* = 0.79)	185 ± 61 (*p* = 0.23)	176 ± 62 (*p* = 0.78)	170 ± 60 (*p* = 0.62)
20 W	24 W	28 W	32 W	36 W
169 ± 59 (*p* = 0.62)	173 ± 62 (*p* = 0.57)	167 ± 65 (*p* = 0.89)	162 ± 59 (*p* = 0.33)	168 ± 62 (*p* = 0.96)
40 W	44 W	48 W	52 W	∆SFCT
164 ± 62 (*p* = 0.79)	171 ± 62 (*p* = 0.90)	167 ± 62 (*p* = 0.73)	162 ± 58 (*p* = 0.40)	−5 ± 37

* Results reported as mean ± SD. *p*-values shown refer to the comparison with the previous time point using paired samples test after performing Bonferroni correction.

**Table 5:** Changes in Retinal Fluid Distribution and SD-OCT Biomarkers during Follow-Up.

	0 W	52 W	P1 *	P2 **	P3 ***	P4 ****
IRF	10 (30%)	6 (18%)	0.25	0.06 (0.32)	0.52 (−0.11)	0.10 (0.30)
SRF	22 (66%)	8 (24%)	<0.01	0.07 (0.31)	0.78 (0.04)	0.16 (0.26)
PED	30 (90%)	24 (72%)	0.54	0.24 (−0.20)	0.84 (0.03)	0.47 (−0.13)
MA	7 (21%)	11 (33%)	0.29	0.13 (0.26)	0.02 (−0.59)	0.90 (0.02)
HRDs	17 (51%)	16 (48%)	0.80	<0.01 (−0.65)	0.06 (−0.32)	0.21 (−0.23)
PCCs	6 (18%)	6 (18%)	1.00	0.57 (−0.10)	0.60 (0.09)	0.11 (0.29)
SHRM	9 (27%)	6 (18%)	0.37	0.39 (0.15)	0.14 (−0.26)	0.45 (−0.14)
SFCT	171 ± 64	162 ± 58	0.40	<0.01 (−0.63)	0.17 (−0.24)	0.49 (−0.13)

* P1: 0 W vs. 52 W, chi-square (χ^2^) test. ** P2: correlation between baseline biomarkers (0 W) and total number of injections needed, Pearson’s correlation coefficient (r). *** P3: correlation between baseline biomarkers (0 W) and BCVA improvement (BCVA 52 W–BCVA 0), Pearson’s correlation coefficient (r). **** P4: correlation between baseline biomarkers (0 W) and CMT reduction (CMT 52 W–CMT 0), Pearson’s correlation coefficient (r). SD-OCT: spectral-domain optical coherence tomography. IRF: intraretinal fluid. SRF: subretinal fluid. PED: pigment epithelial detachment. MA: macular atrophy. HRDs: hyperreflective dots. PCCs: prechoroidal clefts. SHRM: subretinal hyperreflective material. SFCT: subfoveal choroidal thickness. The number of eyes with SRF decreased significantly at 52 W from 22 (66%) to 8 (24%) (*p* < 0.01). There was a negative correlation between the presence of MA at baseline and BCVA improvement (*p* = 0.02, r = −0.59). There was a negative correlation between the presence of HRDs at baseline and total number of injections at 52 W (*p* < 0.01, r = −0.65). There was a negative correlation between baseline SFCT and number of injections at 52 W (*p* < 0.01, r = −0.63). There was no correlation between baseline biomarkers and reduction in CMT at 52 W.

**Table 6:** Previously published papers regarding outcomes of IVF in eyes with previously treated nAMD.

Study	Year	n	Follow-Up Time	Main Finding
Grimaldi et al. [45]	2023	26	30 weeks	IVF improves anatomical outcomes while preserving visual acuity.
Pandit et al. [46]	2023	218	6 months	
Ng et al. [47]	2024	63	6 months	
Kataoka et al. [48]	2024	130	6 months	

IVF: intravitreal faricimab. nAMD: neovascular age-related macular degeneration.

## Data Availability

The original data analyzed in this paper can be provided upon request from the authors.
